# Emotion Regulation Predicts Depressive Symptoms in Adolescents: A Prospective Study

**DOI:** 10.1007/s10964-023-01894-4

**Published:** 2023-11-20

**Authors:** Gyöngyi Kökönyei, Lilla Nóra Kovács, Judit Szabó, Róbert Urbán

**Affiliations:** 1 Institute of Psychology, ELTE Eötvös Loránd University, Budapest, Hungary; 2 NAP3.0-SE Neuropsychopharmacology Research Group, Hungarian Brain Research Program, Semmelweis University, Budapest, Hungary; 3 Department of Pharmacodynamics, Faculty of Pharmacy, Semmelweis University, Budapest, Hungary; 4 National Institute of Criminology, Budapest, Hungary

**Keywords:** Adolescence, Depressive symptoms, Prospective study, Emotion regulation, Latent classes

## Abstract

Emotion regulation as a proximal factor has been linked with depressive symptoms. However, studies have mainly focused on a limited number of strategies and have mostly been cross-sectional in design. This is particularly evident when examining the protective effects of adaptive strategies. This study aimed to investigate the prospective relationship between putatively adaptive and maladaptive emotion regulation strategies and depressive symptoms among adolescents. Additionally, a person-oriented approach was applied to identify latent classes of adolescents based on their depressive symptoms and compared these classes in terms of their adaptive and maladaptive strategies. Two waves of data from a prospective study, which included 1371 youth (mean age: 15.66 years; SD = 0.49 years; 55.1% girls), were analysed. The two points of data collection were spaced approximately half a year apart. Depressive symptoms were measured using the Center for Epidemiologic Studies Depression Scale, and putatively adaptive and maladaptive emotion regulation strategies were assessed with the Cognitive Emotion Regulation Questionnaire. Seven strategies (acceptance, positive refocusing, positive reappraisal, putting into perspective, self-blame, rumination, and catastrophizing) were categorised into adaptive and maladaptive factors using exploratory structural equation modeling. After controlling for gender, age, and depressive symptoms at Time 1, both maladaptive and adaptive emotion regulation strategies at Time 1 predicted depressive symptoms at Time 2. Three subgroups emerged based on the intensity of depressive symptoms across the waves: the stable low, stable moderate, and stable high depressive symptom groups. The use of maladaptive emotion regulation strategies (such as rumination, self-blame, and catastrophizing) at Time 1 was more pronounced in the stable moderate and high symptom groups compared to the stable low depressive symptom group. The comparable prospective associations between putatively adaptive and maladaptive strategies with symptoms suggest the need to identify factors that may mitigate the negative impact of maladaptive emotion regulation and/or promote adaptive emotion regulation to buffer the effects of everyday stressors.

## Introduction

Adolescence, as a developmental phase, is often linked with the onset of some psychopathologies (Luciana, 2013). In fact, one-third of 10–19-year-olds are at risk of developing clinical depression, and the lifetime prevalence of major depressive disorder within this age range is 19% (Shorey et al., 2022). Furthermore, the prevalence of elevated depressive symptoms (which increases the risk of depression) and depressive disorders has increased in recent decades. For example, there was an increase in depressive disorders among 12–20 year-olds from 2005 to 2014 (Mojtabai et al., 2016), and when comparing the period of 2001–2010 with 2011–2020, the prevalence of elevated depressive symptoms (the risk of depression) increased from 24% to 37% in the 10–19 age group (Shorey et al., 2022). Young adults who have persistent depressive symptoms since adolescence might experience negative consequences in adulthood, such as increased risk for self-harm with suicidal intent, more functional impairments in different life domains (Weavers et al., 2021), or more physical health problems (Wickrama et al., 2009). Investigating the longitudinal trajectories of depressive symptoms a meta-analysis (Shore et al., 2018) showed that a large proportion of adolescents experience no/low or moderate symptoms during development. However, for some adolescents, symptoms of depression are stable at high levels or increase over time. While there is a substantial body of evidence suggesting that emotion regulation predicts depression symptoms over time during adolescence (Cavicchioli et al., 2023), our understanding of this association remains limited. Much of the research has primarily focused on one or a few putatively maladaptive strategies, thus restricting our insight. Only a handful of longitudinal studies have explored the prospective association between emotion regulation and depressive symptoms, particularly examining both adaptive and maladaptive strategies. To address this gap in the literature, this study aims to investigate various emotion regulation strategies in the context of depressive symptoms in adolescents using a longitudinal design.

### Emotion Regulation

The ability to regulate emotions, which involves altering the timing, intensity, duration, valence, and/or expression of emotions to meet the goals or situational demands (Gross, 1998; Thompson, 1994), is particularly critical during this developmental period. Ecological momentary assessment studies (Reitsema et al., 2022) and longitudinal studies (Larson et al., 2002) show that more intense negative affects are experienced during adolescence than in childhood. The fluctuation of sad mood is also higher in adolescence than in childhood (Reitsema et al., 2022). These changes in emotional dynamics are likely linked to new challenges such as autonomy and identity formation (Alonso-Stuyck et al., 2018), risk-taking (Icenogle & Cauffman, 2021), an increased need for social acceptance and approval (Somerville, 2013), the emergence of romantic feelings (Collins et al., 2009), and other aspects of intrapersonal and interpersonal functioning in adolescents.

### Rumination

One particular emotion regulation strategy that has received significant attention is rumination, which has been linked to depression and depressive symptoms in adolescents (Schafer et al., 2017), after the development of the Response Style Theory (RST) (Nolen-Hoeksema, 1991). Longitudinal studies have suggested that rumination (defined as having non-productive, recurrent, and distress-focused thoughts (Nolen-Hoeksema, 1991)) predicts the development of psychopathology in adolescents (Hankin, 2008), particularly depression (Abela & Hankin, 2011; Rood et al., 2009). While ruminating, negative or neutral aspects of past or present events are processed in a way that, in the absence of cognitive control (Joormann, 2010), can easily maintain or even amplify negative emotions. This being one of the possible mechanisms for the development of depressive symptoms/depression (Watkins & Roberts, 2020). Although the relationship between rumination and the occurrence of different psychopathologies or symptoms is fairly consistent among both adults and adolescents (Aldao et al., 2010; Cludius et al., 2020; Schafer et al., 2017), other emotion regulation strategies should also be considered (Aldao et al., 2016). Indeed, research has shown that several emotion regulation strategies are worth exploring within the context of internalizing symptoms, such as depression in adolescents (Garnefski et al., 2005; Schafer et al., 2017). While it is important to consider that context plays a significant role in emotion regulation, when employing a strategy-based approach, commonly used strategies are typically categorized as either adaptive or maladaptive based on their associations (Aldao et al., 2010). Strategies such as rumination, avoidance, and suppression are potentially maladaptive since they are positively correlated with psychopathological symptoms. In contrast, other strategies like reappraisal, problem-solving, and acceptance are labelled as adaptive because they show a negative relationship with psychopathological symptoms (Aldao et al., 2010).

### Distraction and Problem-solving

Notably, Response Style Theory (RST) emphasised the importance of various emotion regulation strategies in depression thirty years ago. Specifically, RST (Nolen-Hoeksema, 1991) posits two additional cognitive response styles in relation to depression: distraction and problem-solving. Similar to rumination, distraction (defined as the effortful or passive diversion of attention towards neutral or positive stimuli (Webb et al., 2012)) and problem-solving (defined as indirect attempts to regulate emotions, such as planning the course of action to handle a situation or problem (Aldao et al., 2010)) can be seen as emotion regulation strategies due to their ability to change the trajectory of emotions (Gross, 1998). In RST, both distraction and problem-solving (categorised as adaptive according to the strategy-based approach) were expected to decrease depressive symptoms. Indeed, there are longitudinal findings that support the original theory among adolescents (Hilt et al., 2010), although contradicting results also exist (van Ettekoven et al., 2020). While problem-solving may be effective in down-regulating high-intensity daily negative emotions, according to a recent finding from an experience sampling method (ESM) study in adolescents (Lennarz et al., 2019), the use of this strategy may be limited and cannot be employed in many situations (e.g., peer rejection, poor body-image, recalled negative events from the past) that trigger or are associated with depressive mood.

Regarding distraction, a meta-analysis of studies on emotion regulation strategies employing experimental design highlights the effectiveness of positive refocusing (i.e., thinking about positive things instead of negative events (Garnefski et al., 2001)), as a form of the distraction (Webb et al., 2012). Self-reported positive refocusing was associated with lower depressive symptoms in adolescents (Garnefski & Kraaij, 2016). However, if distraction is habitually used to avoid unwanted memories and related emotions and thoughts (a phenomenon known as experiential avoidance (Hayes et al., 1996)), it might be associated with poor mental health in adults (Wolgast & Lundh, 2017) and youth (Szemenyei et al., 2020).

### Self-blame and Catastrophizing

Besides rumination, other potentially maladaptive emotion regulation strategies, such as self-blame (attributing negative events to oneself), have also been linked to depressive symptoms in adolescents (Garnefski & Kraaij, 2016), supporting cognitive models of depression (Abramson et al., 1978; Beck, 1987) in which self-blame is related to negative self-evaluation. The impact of self-blame on mental health can be particularly important in the case of real or anticipated negative events. During adolescence, peer acceptance is of great importance, and experienced or anticipated rejection and reactions to it, including attempts to regulate emotions, may be linked to the development of depression. For instance, those who tended to blame themselves for being rejected in hypothetical scenarios had more depressive symptoms after 14 months (Zimmer-Gembeck et al., 2016). Another maladaptive emotion regulation strategy is catastrophizing, which involves magnifying and exaggerating the potential negative impact and consequences of an event, repeatedly returning to these thoughts, and/or anticipating the worst possible (almost catastrophic) outcome (Beck, 1987; Ellis, 1962). This strategy has been associated with depressive symptoms in both school and clinical samples (Ding et al., 2021), which is consistent with cognitive models of depression (Abramson et al., 1978; Beck, 1987).

### Reappraisal and Acceptance

Regarding putatively adaptive strategies, a meta-analysis on self-report cross-sectional studies in youth (Schafer et al., 2017) found that problem-solving, positive reappraisal (which involves reinterpreting the meaning of a stimulus to reduce its personal significance (Gross & John, 2003)), and the acceptance of emotions (without judging them as aversive, (Hayes & Wilson, 2003)) were significantly negatively associated with depressive symptoms. The magnitude of the relationship was comparable to the effects between maladaptive strategies and depressive symptoms. In contrast, a recent meta-analysis found that the impact of maladaptive emotion regulation strategies at baseline on the development of psychopathological symptoms was larger than that of adaptive emotion regulation strategies in children and adolescents (Cavicchioli et al., 2023). Specifically, only maladaptive emotion regulation strategies at Time 1 - but not adaptive emotion regulation strategies - were significant predictors of internalizing symptoms. However, in everyday life, an ESM study (Lennarz et al., 2019) showed that problem-solving, reappraisal, and acceptance were more effective in reducing negative emotions than rumination among adolescents.

### Association between Adaptive and Maladaptive Strategies

Considering that individual may use various strategies within and across situations, it is essential to understand the relationships between different emotion regulation strategies. Some studies in children and adolescents (Abela et al., 2007; Hilt et al., 2010) utilizing the RST have adopted a ratio approach to assess the relative use of each strategy – both maladaptive and adaptive – compared to one other. The ratio score is computed by dividing an individual’s mean rumination score by the sum of the mean problem-solving and mean distraction scores. This method allows for the differentiation between individuals who frequently employ multiple strategies, including rumination, distraction, and problem-solving, and those who predominantly rely on rumination alone. Studies employing the ratio score have demonstrated its ability to predict an increase in depressive symptoms over time (Hilt et al., 2010).

To determine whether putatively adaptive or maladaptive strategies are equally important for the development of depressive symptoms, it is necessary to measure the use of several emotion regulation strategies simultaneously (Aldao et al., 2016). Additionally, positive correlations between putatively adaptive and maladaptive strategies are often observed (Kokonyei et al., 2019), suggesting that individuals may use a wide range of strategies across situations. Adolescents, for instance, may employ acceptance as a response to stressful events that trigger less intense emotions. However, they might opt for other strategies like rumination or distraction when dealing with more intense negative emotions (Lennarz et al., 2019). Furthermore, adaptiveness of a given strategy depend on the context as well. For example, a reappraisal of manageable stressors may not promote psychological health (Troy et al., 2013).

## Current Study

**Fig. 1:**
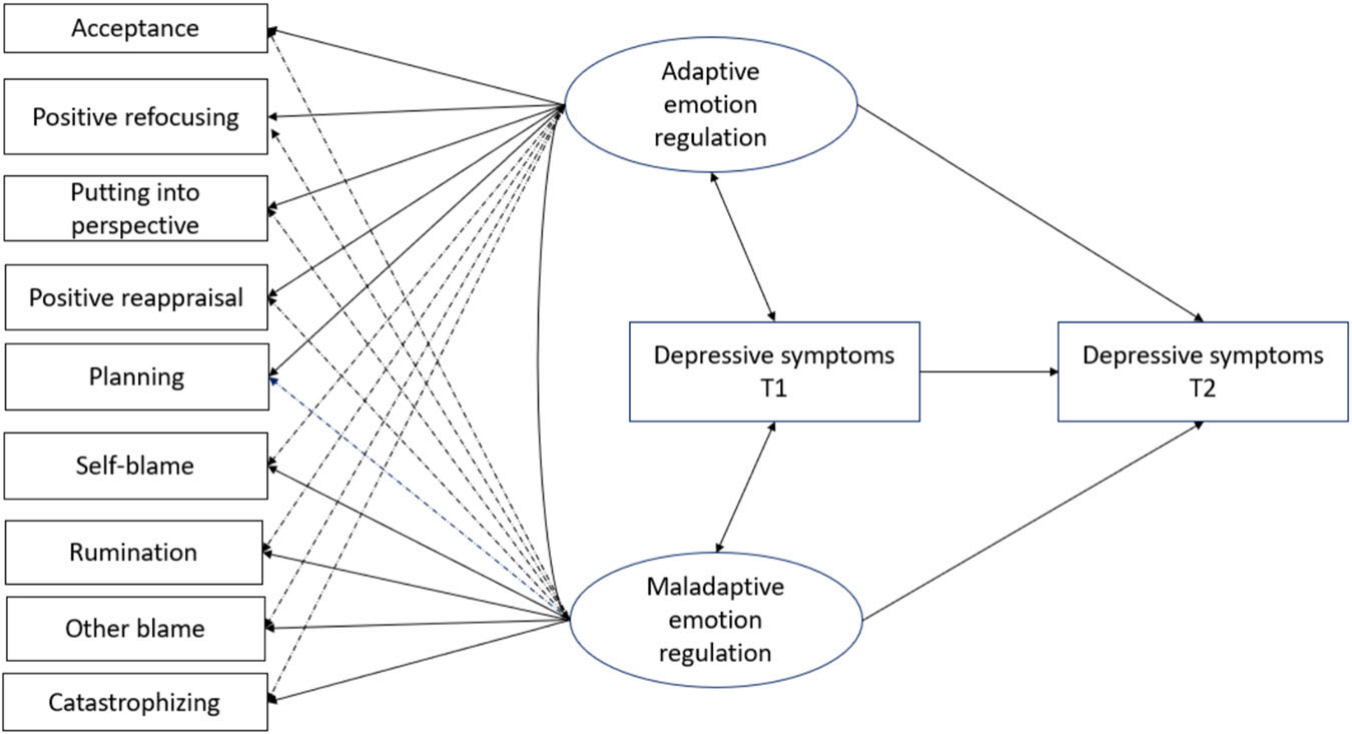
Schematic representation of the measurement model of putatively adaptive and maladaptive emotion regulation strategies and the tested model for explaining depressive symptoms at Time 2. Emotion regulation was measured at Time 1. Regarding the measurement model (i.e., the latent factors of emotion regulation), arrows with solid lines represent main factor loadings, dashed arrows represent cross-loadings

While previous research has shown that emotion regulation strategies are associated with depressive symptoms both concurrently and prospectively, there is limited evidence regarding the emergence of this relationship when assessing both adaptive and maladaptive strategies simultaneously within a single study. This study aims to address this gap by investigating the prospective relationship between several putatively adaptive and maladaptive emotion regulation strategies and depressive symptoms while considering the complexity of interrelation among strategies. Specifically, it was hypothesised that both adaptive and maladaptive emotion regulation at Time 1 would prospectively predict depressive symptoms (Fig. 1). Additionally, the relationship between emotion regulation strategies and depressive symptoms can be further addressed by exploring potential differences in emotion regulation among groups categorized based on depressive symptom patterns across waves of data collection. Therefore, the objective also included to examine how the subgroups of adolescents with various change patterns in depression differed from each other in terms of putatively adaptive and maladaptive strategies.

## Methods

### Sample and Procedure

Data analyzed in the current study was obtained during the second and the third waves of the Budapest Adolescent Smoking Study, a longitudinal research involving high school students (Urban, 2010; Urban & Sutfin, 2010). After obtaining the ethical consent of the Institutional Review Board, 150 secondary schools in Budapest were contacted to get involved in the study that mainly focused on smoking among adolescents. There were 70 schools that agreed to participate, among which schools representing each district were chosen randomly. In case a school did not reply after being contacted three times or refused to participate, another randomly chosen school from the same district was invited. The obtained sample is representative of secondary schools in Budapest in terms of spatial distribution (the 23 districts) and school type (the general secondary schools, vocational secondary schools, and vocational school). One or two classes per school were chosen randomly; thus, 3124 students from 106 classes were invited. Parents received a passive informed consent. Participation was voluntary, the questionnaires were completed during a school class. The final sample at the first wave of data collection comprised 2565 students. There were 2513 ninth-grade students who participated in the second wave, whereas 2370 tenth-grade students participated in the third wave. These dropouts arose mainly due to non-consent or being absent on the day of data acquisition. There were 1857 students involved in both waves, of whom 486 had to be excluded due to missing data on all depression items. Thus, 1371 adolescents were included in the current analysis. The mean age of our sample (as measured at the second wave) was 15.66 years (SD = 0.49 years), 55.1% (N = 756) were girls. Data collection occurred between March and May 2009 for the second wave (Time 1), and between October and December 2009 for the third wave (Time 2). There were 5.9 months that had elapsed between the two points of data collection on average. The study was not preregistered.

### Measures

#### Emotion Regulation

Emotion regulation strategies were measured with the 18-item *Cognitive Emotion Regulation Questionnaire (CERQ-short)* (Garnefski & Kraaij, 2006). Respondents were instructed to state on a five-point Likert scale (ranging from almost never to almost always) how often they tend to react in certain ways to stressful life events. The CERQ-short comprises of nine subscales, each measured by two items: (1) self-blame (attributing the negative event to ourselves), (2) other-blame (attributing the negative event to other people), (3) rumination (repetitively dwelling on a negative event), (4) catastrophizing (magnifying the severity or significance of a negative event), (5) putting into perspective (comparing the event with others leads to the conclusion that it is not the worst, or that it could have been worse), (6) positive refocusing (diverting attention towards unconnected positive events), (7) positive reappraisal (reinterpreting the event in order to see the positive aspects of a negative event), (8) acceptance (the situation or what has happened), and (9) planning (focusing on the course of actions one can do to change or cope with the situation), among which the first four are considered putatively maladaptive, the latter five are considered putatively adaptive responses to a demanding situation. The Hungarian CERQ was used in the current study which demonstrated good psychometric properties in previous studies among adults (Miklósi et al, 2011) and adolescents (Kokonyei et al., 2019). Emotion regulation was measured at Time 1. In the current sample, the Cronbach α values of the subscales ranged from 0.61 to 0.82 (Table 1).

**Table 1:** Means, standard deviations, and effect sizes (Cohen’s d) by gender with reliability of the scales

Scales	Mean (SD)	Boys Mean (SD)	Girls Mean (SD)	t/d	p	Cohen’s d	Cronbach’s alpha
self-blame	4.69 (1.85)	4.53 (1.75)	4.82 (1.92)	2.90	0.004	0.16	0.80
acceptance	5.71 (2.05)	5.80 (2.04)	5.65 (2.05)	1.33	0.182	0.07	0.81
rumination	5.95 (2.03)	5.55 (1.92)	6.27 (2.06)	6.59	<0.001	0.36	0.71
positive refocusing	4.78 (2.01)	4.73 (1.93)	4.82 (2.07)	0.80	0.426	0.04	0.82
planning	6.24 (1.96)	6.17 (1.91)	6.29 (2.00)	1.08	0.281	0.06	0.68
positive reappraisal	6.11 (1.98)	6.15 (1.94)	6.08 (2.02)	0.71	0.480	0.04	0.61
putting into perspective	5.18 (1.93)	5.02 (1.80)	5.32 (2.01)	2.94	0.003	0.16	0.70
catastrophising	4.16 (1.96)	3.93 (1.80)	4.35 (2.08)	3.91	<0.001	0.21	0.81
other-blame	4.11 (1.72)	4.39 (1.81)	3.89 (1.62)	5.28	<0.001	0.29	0.81
CES-D T1	15.50 (10.16)	13.93 (9.48)	16.77 (10.52)	5.20	<0.001	0.28	0.90
CES-D T2	16.21 (9.93)	14.00 (9.12)	18.01 (10.20)	7.68	<0.001	0.41	0.90

N_boys_ = 615; N_girls_ = 756; *CERQ* Cognitive Emotion Regulation Questionnaire; *CES-D* The Center for Epidemiologic Studies Depression Scale

#### Depressive Symptoms

*The Center for Epidemiologic Studies Depression Scale* (Radloff, 1977) is a widely used self-report measure for depressive symptoms in adult and adolescent community samples. Participants are asked to rate how often they felt certain affect states over the past week on a four-point Likert scale that ranges from 0 (rarely or none of the time; less than one day) to 3 (most or all of the time; five to seven days). The measure contains 20 items, the majority of which describe negative affect states (e.g., I felt lonely; I felt sad), while four items are reversed and describe positive affect states (e.g., I felt hopeful about the future). Depressive symptoms, the predicted outcome, were assessed at both Time 1 and 2. In previous studies involving adult samples, the Hungarian CES-D has been demonstrated as a reliable tool for assessing depressive symptoms (e.g., Demetrovics, 2007; Kovacs et al., 2021). While the CES-D is widely used to assess adolescents in several languages, there is less experience with this measure among Hungarian adolescents. However, in this sample, the Hungarian CES-D demonstrated excellent reliability. Cronbach α was 0.90 at both Time 1 (wave 2) and Time 2 (wave 3), suggesting that the Hungarian version is also a reliable scale.

#### Data Analytic Plan

Data were analyzed using SPSS 28.0 (IBM SPSS, IBM Corp., Armonk, NY) and Mplus 8.8 software packages (Muthén & Muthén, 1998–2007). First step was to test whether the nine strategies of CERQ could be grouped into adaptive and maladaptive emotion regulation factors. Based on previous findings, Exploratory Structural Equation Modeling (ESEM) was chosen instead of confirmatory factor analysis (CFA), as the correlations between the putatively adaptive and maladaptive strategies have been found to be positive in many cases (Kokonyei et al., 2019) suggesting that individuals may use a wide range of strategies. In addition, ESEM provides the possibility to build a model in which cross-loadings are allowed (i.e., targeted but not fixed at zero) across factors. The maximum likelihood robust (MLR) parameter estimates with standard errors and chi-square test statistics were used that were robust to non-normality and non-independence of observation (Muthén & Muthén, 1998–2007). Since units were classes in the current study, the nonindependence of observations was controlled for in the analysis.

In the next step, structural equation modeling (SEM) was used to examine whether adaptive and maladaptive emotion regulation defined as latent factors allowing for cross-loadings (as in the first step) at Time 1 predicted depressive symptoms at Time 2, controlling for gender, age and depressive symptoms of Time 1. Depressive symptoms were defined as observed variables in the model.

For both the measurement model and the longitudinal model, model fit was evaluated by inspecting the fit indices: the Root Mean Squared Error of Approximation (RMSEA) and the significance of its value (cfit), the Comparative Fit Index (CFI), the Tucker-Lewis Index (TLI) and Standardized Root Mean Square Residual (SRMR). The model fits well if the RMSEA is below 0.05 (a value above 0.10 indicates poor fit), and cfit is non-significant. Regarding the CFI and TLI, values higher than 0.90-0.95 indicate an acceptable-good fit (Brown, 2006). As for the SRMR, a value below 0.08 is considered a good fit (Kline, 2016).

In the next step, latent class analysis was used to identify homogenous subgroups (latent classes) of participants based on their depressive symptoms scores at Time 1 and Time 2. The optimal number of classes were determined by considering models with a different number of latent classes using the Akaike Information Criterion (AIC), Bayesian Information Criterion (BIC), Sample Size Adjusted Bayesian Information Criterion (SSA-BIC), Lo-Mendel-Rubin Adjusted Likelihood Ratio Test (LMRT). Lower values of AIC, BIC, SSA-BIC indicate better fit and non-significant LMRT shows that the involvement of an additional latent class would have not increased the fit of the model. The index of Entropy with higher values (e.g., closer to 1) reflects a more accurate classification of the participants (e.g., values at around 0.8 represent high entropy) (Clark & Muthén, 2009). Then multinominal logistic regression analysis was used to explore the relationship between the most likely latent class membership and covariates (gender, age, latent factors of adaptive and maladaptive emotion regulation indexed by factor scores) using the 3-step method.

## Results

### Descriptive Data

The final sample included in the present analysis comprised 1371 students (all were Hungarian, N = 756 (55.1%) girls) who attended secondary schools in the capital city of Hungary. Three-fifths of students (61.5%) attended secondary grammar schools and 39.8% attended vocational high schools. Mothers of around three quarters of students (77.4%) had a high school diploma or a university degree. Most of the students (63.2%) lived in their intact families (i.e. in nuclear families with both parents present).

Descriptive data for emotion regulation (ER) strategies and depressive symptoms are presented in Table 1. In both waves, girls reported more depressive symptoms. Regarding the ER strategies, girls had higher scores on self-blame, rumination, putting into perspective, catastrophizing, and other-blame.

For descriptive purpose only, and based on previous research on adolescent depression (e.g. Rushton et al., 2002), a score of 24 was employed as the cutoff (≥ 24) on CES-D (refer to Shore et al., 2018) and 21.4% (N = 294) of the participants at Time 1 and 21.9% (N = 300) of the participant at Time 2 scored 24 or above, and 12.3% (N = 168) were in the risk group in both waves.

In general, ER strategies were correlated with each other, and the correlation coefficients ranged between −0.15 and 0.49 (Table 2). Depressive symptoms in both waves were positively associated with the putatively maladaptive ER strategies, namely with self-blame, rumination, and catastrophizing (correlation ranges between r = 0.23 to 0.44), and negatively with the putatively adaptive ER strategies, namely acceptance, positive refocusing, positive reappraisal, putting into perspective (correlation ranges between r  = −0.01 to −0.14). Age had a weak relationship with depressive symptoms, but it was unrelated to the use of ER strategies.

**Table 2:** Correlations between depressive symptoms and cognitive emotion regulation strategies

	2	3	4	5	6	7	8	9	10	11	12
1. Self-blame	0.21^**^	0.39^**^	−0.11^**^	0.29^**^	0.25^**^	0.04	0.36^**^	−0.15^**^	0.30^**^	0.23^**^	−0.03
2. Acceptance	1	0.25^**^	0.10^**^	0.20^**^	0.41^**^	0.24^**^	0.05	−0.07^*^	−0.06^+^	−0.05	−0.03
3. Rumination		1	−0.15^**^	0.38^**^	0.24^**^	0.10^**^	0.48^**^	0.06^+^	0.29^**^	0.23^**^	−0.03
4. Positive refocusing			1	0.10^**^	0.19^**^	0.40^**^	−0.14^**^	0.14^**^	−0.14^**^	−0.14^**^	0.00
5. Planning				1	0.43^**^	0.29^**^	0.17^**^	0.07^*^	0.03	0.01	−0.01
6. Positive reappraisal					1	0.32^**^	0.03	−0.03	−0.01	−0.04	0.04
7. Putting into perspective						1	−0.04	0.13^**^	−0.06^+^	−0.05	0.02
8. Catastrophizing							1	0.21^**^	0.44^**^	0.33^**^	0.02
9. Other-blame								1	0.09^**^	0.05^+^	0.01
10. CES-D Time 1									1	0.61^**^	0.11^**^
11. CES-D Time 2										1	0.06^+^
12. age											1

*CERQ* Cognitive Emotion Regulation Questionnaire; *CES-D* The Center for Epidemiologic Studies Depression Scale

^+^*p* < 0.05; *p < 0.01; **p < 0.001

**Table 3:** Standardized factor loadings of the final two-factor ESEM model comprising seven emotion regulation strategies

Strategies	Adaptive emotion regulation	Maladaptive emotion regulation
Acceptance	0.521	0.128
Positive refocusing	0.311	−0.268
Positive reappraisal	0.746	0.066
Putting into perspective	0.437	−0.041 (ns)
Self-blame	0.142	0.514
Rumination	0.108	0.709
Catastrophizing	−0.182	0.712

All factor loadings are significant, except one. ns: non-significant. N = 1362, nine students did not answer the emotion regulation items.

### Measurement Models of Emotion Regulation Strategies

First step was to test whether the nine strategies constitute two factors. Based on the literature (Garnefski et al., 2001), self-blame, other-blame, rumination, and catastrophizing are putatively maladaptive strategies, while acceptance, positive refocusing, planning, reappraisal, and putting into perspective are putatively adaptive strategies. In the ESEM analysis, cross-loadings were allowed (targeted, but not forced to be zero). The first ESEM model comprising all the nine strategies resulted in a poor fit (χ^2^ = 347.3, df=19, p < 0.001, RMSEA: 0.113 [0.102-0.123] cfit: <0.001; CFI: 0.804, TLI:0.628, SRMR: 0.056), and the analysis pointed out that other-blame did not load on any factors. Therefore, in the repeated analysis the other-blame subscale was omitted and based on the modification indices of the first ESEM model error covariances between the refocusing and putting into perspective subscales were added to the model. The fit indices of the second model improved and the model fit become already acceptable (χ^2^ = 76.8, df=12, p < 0.001, RMSEA: 0.063 [0.050-0.077] cfit: p = 0.050; CFI: 0.959, TLI:0.905, SRMR: 0.026); however, factor loadings of the planning subscale suggested that this factor can be labelled neither adaptive nor maladaptive (since it loaded on both factors positively (0.469 and 0.261), and the difference between the two factor loadings was considerably smaller compared to the other items). Thus, in the final model seven strategies (namely acceptance, positive refocusing, positive reappraisal, putting into perspective, self-blame, rumination, and catastrophizing) were grouped into adaptive and maladaptive emotion regulation factors. Factor loadings are presented in Table 3. The final model resulted in an excellent fit (χ^2^ = 28.6, df=7, p < 0.001, RMSEA: 0.048 [0.030-0.066] cfit: 0.550; CFI: 0.981, TLI: 0.944, SRMR: 0.018). The correlation between the two factors was 0.22. These two latent factors were used in the next step to explain depressive symptoms at Time 2.

### Prospective Association between Adaptive and Maladaptive Emotion Regulation Strategies with Depressive Symptoms

**Fig. 2:**
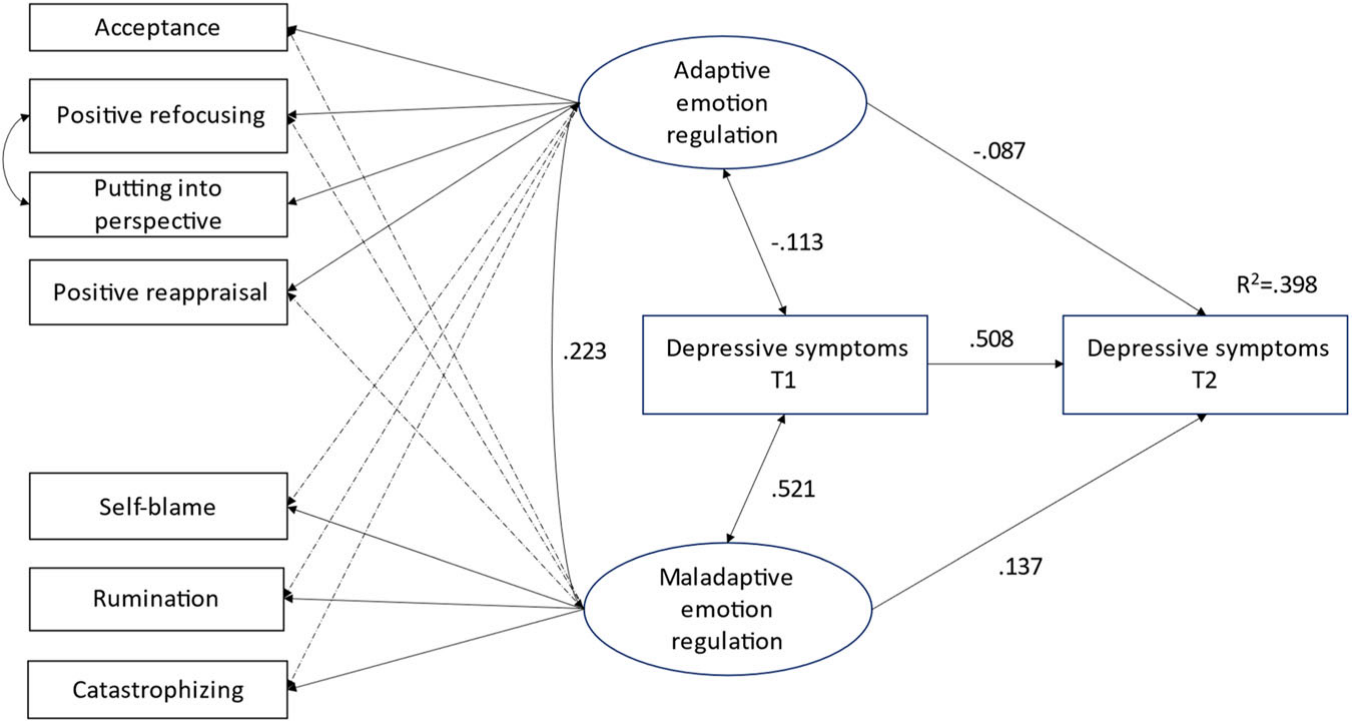
N = 1371. Standardized coefficients are presented. All presented paths shown are significant. Dashed lines represent significant cross-loadings. Gender and age were controlled for in the model. Exploratory Structural Equation Modeling (ESEM) was used to define the Adaptive and Maladaptive emotion regulation latent factors. Depressive symptoms at Time 1 (T1) and Time 2 (T2) were observed variables

In the next step, the model examining the prospective association between adaptive and maladaptive ER at Time 1 and depressive symptoms at Time 2 – while taking into account depressive symptoms at Time 1 – was estimated. Gender and age were controlled for in this model (Table 4). The covariance between depressive symptoms at Time 1 and between adaptive and maladaptive ER were also estimated in the model (see Fig. 2).

**Table 4:** Correlation matrix of emotion regulation and depressive symptoms

	Adaptive emotion regulation	Maladaptive emotion regulation	Depressive symptoms Time 1	Depressive symptoms Time 2	Gender
Maladaptive emotion regulation	0.219^***^				
Depressive symptoms at Time 1	−0.111^*^	0.529^***^			
Depressive symptoms at Time 2	−0.114^*^	0.405^***^	0.608^***^		
Gender	−0.007	0.169^***^	0.139^***^	0.201^***^	
Age at Time 1	0.010	−0.012	0.105^***^	0.063^*^	−0.127^***^

N = 1371

*p < 0.05; ***p < 0.001

The result showed that even controlling for depressive symptoms at Time 1, both maladaptive (β = 0.137, p = 0.001) and adaptive (β =−0.087, p = 0.013) ER at Time 1 predicted depressive symptoms at Time 2. The overall model fitted the data well (χ = 104.2, df=27, p < 0.001, RMSEA: 0.046 [0.037-0.055]; cfit: 0.765; CFI: 0.966, TLI: 0.932) and explained 39.8% from the total variance of depressive symptoms at T2. Examining the absolute values of the coefficients, the concurrent association between maladaptive emotion regulation and symptoms is stronger than the association between adaptive emotion regulation and symptoms (Wald test = 69.68, df = 1, p < 0.001). Regarding the prospective association, however, we did not find a difference between the magnitudes of the two coefficients (Wald test = 1.82, df = 1, *p* = 0.177). In other words, adaptive and maladaptive ER were equally predictive of depressive symptoms at Time 2.

### Latent Class Analysis

Based on the fit indices of the LCA, the 3-class solution proved to be the best model (see Table 5). The LMRT of the 4-class model was no longer significant (p = 0.14), suggesting the three-cluster over the four-cluster solution.

**Table 5:** Fit indices for the latent class analysis of depressive symptoms at Time 1 and Time 2

	AIC	BIC	SSA-BIC	Entropy	LMRT	*p*
2 class model	19739.3	19755.9	19753.6	0.820	674.8	<0.001
**3 class model**	**19548.4**	**19600.7**	**19568.9**	**0.827**	**188.2**	**<0.001**
4 class model	19514.3	19582.2	19540.9	0.830	38.4	0.1403

*AIC* Akaike Information Criteria; *BIC* Bayesian Information Criteria; *SSA-BIC* Sample Size Adjusted Bayesian Information Criteria; *LRT* Lo-Mendel-Rubin Adjusted Likelihood Ratio Test. The selected model is boldfaced

**Fig. 3:**
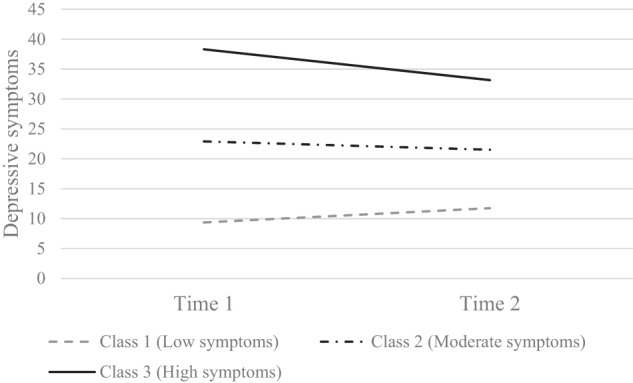
The three latent classes of depressive symptoms (N = 1371)

Figure 3 shows the three classes: Class 1 (N = 870, 63.5%) had low scores at both waves (M_T1_ = 9.36, SD_T1_ = 5.02; M_T2_ = 11.57, SD_T2_ = 7.52, stable low depressive symptoms group), Class 2 (N = 403, 29.4%) had moderate scores at both waves (M_T1_ = 22.91, SD_T1_ = 5.02; M_T2_ = 21.52, SD_T2_ = 7.52, stable moderate depressive symptoms group), and Class 3 (N = 98, 7.1%) had high scores at both waves (M_T1_ = 38.31, SD_T1_ = 5.02; M_T2_ = 33.16, SD_T2_ = 7.52, stable high depressive symptoms group) (see Fig. 3).

### Multinomial Logistic Regression to Predict Class Membership

In the next step, a multinomial logistic regression analysis was performed (see Table 6), in which auxiliary variables, such as gender, age, and adaptive and maladaptive ER strategies (as latent factors indexed by factor scores) predicted class membership. Compared to the low symptoms group, increased use of maladaptive ER strategies and decreased use of adaptive ER strategies were associated with being in moderate and high symptoms groups.

**Table 6:** Multinomial logistic regression analysis to predict moderate and high depressive symptoms groups

Predictors	Stable moderate depressive symptoms OR [95% CI]	Stable high depressive symptoms OR [95% CI]
Maladaptive emotion regulation	3.34 [2.41–4.62]^***^	8.27 [5.53–12.36]^***^
Adaptive emotion regulation	0.62 [0.48–0.79]^***^	0.40 [0.28–0.57]^***^
Age at Time 1	1.91 [1.39–2.63]^***^	2.82 [1.62–4.90]^***^
Gender (Ref. group: boys)	1.53 [1.12–2.08]^**^	2.36 [1.14–4.90]^*^

Reference group is the stable low depressive symptoms class. Maladaptive and adaptive emotion regulations are used as factor scores in the analysis. N = 1362

*p < 0.05, **p < 0.01, ***p < 0.001

## Discussion

Approximately one in three adolescents experiences elevated depressive symptoms (Shore et al., 2018), and some of them also report short- or long-term negative consequences (Weavers et al., 2021). Emotion regulation has been extensively studied as a proximal factor associated with depressive symptoms. However, most of these studies have focused on only a limited number of strategies. Moreover, the majority of research has been cross-sectional, especially in the context of examining the protective effects of adaptive strategies. Hence, this study aims to investigate how various emotion regulation strategies, including both adaptive and maladaptive ones, predict depressive symptoms over time. The longitudinal design of the study also enables to explore differences in emotion regulation strategies among subgroups of adolescents based on their patterns of depressive symptoms over time. Generally, this study found that both maladaptive and adaptive emotion regulation strategies predicted depressive symptoms cross-sectionally and prospectively in adolescents. While the concurrent association between maladaptive emotion regulation strategies and depressive symptoms was stronger compared to the concurrent association between adaptive emotion regulation strategies and depressive symptoms, the adaptive strategies had comparable effect on depressive symptoms prospectively. In addition, three subgroups were identified based on the intensity of depressive symptoms across waves which differed from each other on both the putatively adaptive and maladaptive strategies measured at Time 1.

Existing research, both cross-sectional (Schafer et al., 2017) and prospective (Cavicchioli et al., 2023), suggests that inter-individual differences in emotion regulation explain depressive symptoms during adolescence, and the current study supports this well-established association. As stressors and the context in which emotions are regulated can considerably vary, the efficacy and adaptiveness of certain strategies may also differ (Sheppes & Meiran, 2007). Therefore, it is not surprising that most strategies loaded positively on both factors in the analysis. However, the magnitude of factor loadings reflected the supposed nature of the strategies. For example, positive reappraisal had a considerably higher factor loading on the adaptive emotion regulation factor (0.75) than on the maladaptive emotion regulation factor (0.07). Conversely, rumination had a stronger loading on the maladaptive factor than on the adaptive factor (0.71 vs. 0.11). The stark contrast in factor loadings in these cases suggests that reinterpreting stressful events in a more positive light (as defined in the CERQ) is a more adaptive approach, while dwelling on stressful events through rumination is a less adaptive one. However, there are situations where reinterpreting an event or situation in a positive manner can hinder the attainment of long-term goals (Aldao et al., 2015). For example, consistently perceiving additional work as a sign of trust could negatively impact one’s ability to spend quality time with loved ones and, in turn, have an adverse effect on mental health. Similarly, from some perspectives, ruminating over events that indicate a discrepancy between one’s desired and actual state is a normal process, provided it is time-limited (Koster et al. 2011). Interestingly, positive refocusing, which is akin to distraction, had a negative loading on the maladaptive factor and a positive loading on the adaptive factor, suggesting that individuals who engage in self-blame, rumination, or catastrophizing may be unable to divert their attention from negative stimuli, both internal and external. In many cases, diverting attention from negative events, such as a classmate being unkind, is beneficial because it prevents individuals from falling into a negative spiral. However, the low factor loadings of positive refocusing on the adaptive and maladaptive factors (+0.311 and −0.268, respectively) suggest that the adaptiveness of this strategy is highly context dependent. For example, if positive refocusing or distraction serves as a means of avoiding internal experiences, a phenomenon referred to as experiential avoidance (Hayes et al., 1996), it is more likely to become maladaptive, as it could exacerbate negative emotions. On the other hand, catastrophizing is likely to be maladaptive regardless of the context, as it had a negative loading on adaptive strategies (−0.18) and a positive loading on maladaptive strategies (0.71).

Consistent with previous meta-analytic results in studies involving adults or adolescents (Aldao et al., 2010; Schafer et al., 2017), the current study also found that maladaptive emotion regulation strategies are strongly associated with depressive symptoms concurrently. However, the main finding is that the prospective association between adaptive emotion regulation strategies and depressive symptoms is comparable to that of maladaptive emotion regulation strategies and depressive symptoms. Therefore, not only the use of maladaptive emotion regulation strategies but also the reduced use of adaptive emotion regulation strategies (reappraisal, putting into perspective, positive refocusing, and acceptance) during stress is an equally important prospective predictor of depressive symptoms. In other words, an increased utilization of these strategies is linked to a reduction in depressive symptoms among youth. The negative relationship between the use of adaptive emotion regulation strategies and depressive symptoms has predominantly been established through cross-sectional studies (Kraft et al., 2023). However, there is a scarcity of longitudinal studies that explore the connection between depressive symptoms and the employment of adaptive strategies (Cavicchioli et al., 2023). Regarding adaptive emotion regulation strategies, it is noteworthy that studies typically measure acceptance, reappraisal, or problem-solving. However, in the present study, rather than measuring problem-solving, we assessed planning, defined as a series of actions one can take to either change or cope with a situation (Garnefski et al., 2001). However, as the definition implies, planning alone does not guarantee problem resolution. The planning process may not necessarily result in a final decision on the course of action or even the implementation of a potential solution. This might explain why planning did not load onto either the adaptive or maladaptive factor.

Repairing mood with different strategies, such as reinterpreting the events or refocusing attention on positive things, could be protective factors. For instance, a longitudinal study among college students found a prospective link between cognitive reappraisal, close peer relationship, and better sociometric position (English et al., 2012). Thus, it is possible that reappraisal protects against mental health problems through improved social functioning. However, it is also possible that a higher level of depressive symptoms may result in less successful reappraisal (Bettis et al., 2019). This notion is supported by a meta-analysis of 13 fMRI studies (mainly with adult participants) that found that depressed patients exhibit reduced activation in the cognitive control network during cognitive reappraisal tasks compared to healthy controls, which suggests that reappraisal may not be effective in depression (Picó-Pérez et al., 2017). Similarly, a study found no relationship between reappraisal success and activity in the dorsolateral and dorsomedial prefrontal cortex in depressed adolescents, which further supports the idea that reappraisal may not be effective in this population (LeWinn et al., 2018).

Furthermore, the choice of a particular strategy is dependent on the situation. For instance, when compared to distraction, cognitive reappraisal is preferred in low-intensity situations according to laboratory studies using strategy choice paradigm (Van Bockstaele et al., 2020). This may be due to it being easier to reinterpret mild negative situations compared to events that trigger strong negative emotions. However, there was no relationship between reappraisal and emotional intensity in an ESM study investigating emotion regulation strategy use in naturalistic settings among adolescents. Instead, that study found that adolescents chose rumination, relaxation, or emotional expression when they experienced intense negative emotions (De France & Hollenstein, 2022). In the context of depression, although depressed people are able to use reappraisal when instructed to do so (e.g., following sadness induction in the lab), they do not appear to choose this strategy spontaneously for reducing their sadness (Ehring et al., 2010). In fact, they usually choose strategies that maintain or even increase their negative mood (Quigley & Dobson, 2014). As reappraisal is related to increased positive affect concurrently and prospectively (lagged-effect) in ESM and daily diary studies (Boemo et al., 2022), a relative lack of reappraisal in everyday life may lead to decreased positive emotions, which in turn could contribute to depressive symptoms. Or alternatively, ineffective use of reappraisal in depression may prevent long-term benefits of this emotion regulation strategy.

Putting into perspective is also considered a kind of appraisal tactic, as it provides a new perspective for evaluating the event. Namely, this strategy refers to comparing the current situation to something worse (e.g., “I tell myself that there are worse things in life”) (Garnefski et al., 2001), thus it is reminiscent of downward counterfactual thinking (e.g., much worse things could have happened) (Davey & McDonald, 2000). Putting into perspective does not require generating new meaning, therefore it may be easier to apply than positive reappraisal in adolescence. However, putting into perspective is used less often by depressed young people than their non-depressed peers in a stressful situation (van den Heuvel et al., 2020).

Various studies have shown the beneficial effects of accepting emotions (Lennarz et al., 2019) and the negative effects of non-acceptance of emotions (including experiential avoidance) (Lydon-Staley et al., 2019; Schafer et al., 2017; Szemenyei et al., 2020). However, the CERQ does not measure this, but the degree of acceptance of the stressful situation itself which is sometimes labelled as resignation (Wolgast et al., 2013). But even so, the CERQ acceptance subscale was more strongly loaded on the adaptive factor in the current study, suggesting that there could be situations in which passively accepting the situation could be an adequate response to stress in adolescents. One study (Lennarz et al., 2019), for example, found that adolescents used acceptance most often in everyday life, and they also found situations with less intense emotions easier to accept. It is worth noting, however, that this research only measured emotion regulation on weekend days in a normative sample. In clinical samples, acceptance appears to be more maladaptive. For example, acceptance of stressful situations (as measured by the CERQ) was positively associated with symptoms among adolescents diagnosed with major depressive disorder (Ding et al., 2021). Based on these results, acceptance of certain situations may be particularly beneficial for mental health if they are otherwise difficult to control and change, in which case acceptance may act as a form of secondary coping (Compas et al., 2001). However, when used in controllable context as a form of resignation, it may work against problem solving.

Although two data points do not allow for trajectories, the latent class analysis method can show some patterns of symptom evolution even with two data points. The analysis indicated that the three groups that emerged differed in the degree of symptom prevalence (low to medium to high), and no groups with increasing or decreasing symptoms were found. In line with previous studies (Shore et al., 2018), most of the students belonged to the stable low symptoms group (approximately 60% of the students), but a substantial proportion of youth (around 30%) reported somewhat elevated symptoms occurrence at both waves (stable moderate symptoms group). Importantly, around 30% of the students were part of the moderate symptoms group, with a mean score around the suggested cutoff (24, Rushton et al., 2022; Shore et al., 2018) for the CES-D 20 item questionnaire. A small percentage (around 7%) of youth, however, were in the stable high symptoms group. These proportions are consistent with the results of a meta-analysis (Shore et al., 2018) or some studies using the CES-D to measure depressive symptoms (Ferro et al., 2015). Conversely, there are some studies (e.g., Rodriguez et al., 2005; Yaroslavsky et al., 2013) that identified a higher proportion of students with stable high symptoms using the CES-D. In line with previous studies (Shore et al., 2018), the current study also found that in both the stable moderate and stable high symptoms groups there were more girls than in the stable low symptoms group.

High symptom groups were reliably replicated across studies (Shore et al., 2018), suggesting that for some youth experiencing high depressive symptoms are relatively stable over time. In the current study, the use of maladaptive emotion regulation strategies (rumination, self-blame, and catastrophising) at Time 1 was more pronounced in this group compared to stable low depressive symptoms group. However, increased use of maladaptive emotion regulation strategies was also present when comparing the stable moderate symptoms group to the stable low symptoms group. The maladaptive strategies measured in this study share the common feature of focusing on the negative aspect of the self, and therefore, could easily co-occur. For instance, self-critical or self-blaming ruminative thoughts can easily lead someone to catastrophize; thus, eliciting them to perceive the situation as much worse than it really is. Biased attention towards, and the increased awareness of, negative external or internal stimuli are both risk and maintenance factors of depression (Disner et al., 2011).

Maladaptive emotion regulation strategies may also contribute to the maintenance of depressive symptoms through other mechanisms. For instance, in a short prospective study excessive reassurance seeking as a maladaptive interpersonal behaviour mediated the association between emotion regulation difficulties and high depressive symptoms (Fearey et al., 2021). Rumination can prolong the emotional or physiological concomitants of stressors even after the stressors have terminated (Radstaak et al., 2011); thus, rumination may contribute to depression via transforming past stressors into present events, in turn reinforcing rumination (Hosseinichimeh et al., 2018). Interestingly, this latter mechanism was found to be stronger in adolescent girls compared to boys (Hosseinichimeh et al., 2018), suggesting that not only increased rumination among girls (Hankin, 2008) but also gender differences in rumination-related mechanisms might contribute to the well-known gender differences in depression.

### Clinical Implications

Overall, based on the proportion of adolescents reporting moderate or high levels of symptoms, monitoring depressive symptoms during adolescence would be important. According to a relatively recent meta-analysis, more than a third (34%) of adolescents aged 10–19 years suffer from mild, albeit often subclinical/subthreshold level of depression (Shorey et al., 2022). A proportion of the group with even moderate symptoms in the current study are likely to meet the criteria for subthreshold depression. Subthreshold depression is a clinically significant condition (Bertha and Balazs, 2013; Noyes et al, 2022), the onset and persistence of which are poorly understood in adolescents.

The findings also suggest that young individuals with moderate to high depressive symptoms may benefit more from intervention programs that focus on emotion regulation. For instance, in the current study students with moderate symptoms had over three times higher odds of engaging in maladaptive emotion regulation strategies compared to those in the low symptom group, whereas students in the high symptom group had more than eight times higher odds of using such strategies. Thus, interventions that help adolescents with moderate to high symptoms to recognize and reduce the use of maladaptive emotion regulation strategies and to adopt more adaptive methods to manage negative emotions are warranted in non-clinical settings as well. For example, cultivating decentering (a metacognitive capacity to observe inner experiences from a distanced perspective) at this stage of development, where there is heightened self-awareness and intense negative emotions, may be effective in ameliorating distress and reducing perseverative thoughts (such as rumination) (Bennett et al., 2021).

Improvements in interventions aimed at regulating emotions within clinical populations are typically associated with decreased psychopathological symptoms (Moltrecht et al., 2021). This suggests that positive changes are feasible in community samples with moderate to high symptoms, if baseline levels of various psychopathological symptoms are considered in these effectiveness trials. Furthermore, the limited impact of interventions designed to enhance emotion regulation in community samples (Eadeh et al., 2021) may be partly attributed to a significant portion of young individuals who do not face substantial challenges in regulating their emotions. Thus, a floor effect may exist within this population (Eadeh et al., 2021).

### Limitations

There are some limitations to the current study. This study only examined urban adolescents in a narrow age range; therefore, generalizability to rural and minority adolescents is limited. As emotion regulation was only measured at the first time point, the scar effect hypothesis, i.e., how increased depressive symptoms affect the use of emotion regulation strategies within individuals across time, could not be tested. A meta-analysis on longitudinal studies in children and adolescents (Cavicchioli et al., 2023) showed that after using the cross-lagged panel model, both adaptive and maladaptive emotion regulation strategies at Time 1 were significant predictors of psychopathological symptoms at Time 2. However, the relationship was mutual due to psychopathological symptoms at Time 1 also predicting adaptive and maladaptive emotion regulation at Time 2. As emotion regulation strategies were not measured at Time 2, mutual relationship could not be tested in the current study. Additionally, only six months passed between the two data collections; therefore, the long-term prediction of symptoms through emotion regulation remains uncertain.

The questionnaire (CERQ) used generally measures emotion regulation strategies in the presence of a stressor. Accordingly, the current study lacked the information on how different characteristics of a given stressful situation (or a life event), such as the intensity of emotions associated with it and its controllability, influence the use of emotion regulation strategies (De France & Hollenstein, 2022; Lennarz et al., 2019). Additionally, it should be noted that the CERQ does not assess particular strategies, including expressive suppression, behavioural or experiential avoidance, which research has linked to psychopathological symptoms in adolescents (Kraft et al., 2023). Regarding avoidance, it has been proposed that dissociation could potentially be an automatic mechanism that contributes to the avoidance of emotional experiences (Cavicchioli et al., 2021).

There are also some findings suggesting that emotion regulation should be assessed in an emotion-specific manner (De France & Hollenstein, 2022). Furthermore, using a specific strategy does not guarantee the success of regulation. It can happen that even after using adaptive strategies, the emotion remains dysregulated (Jazaieri et al., 2013), which cannot be measured by administering the CERQ.

Current study focused on stress-related emotion regulation, but a lack of ability to generate and maintain positive emotions (Nelis et al., 2015) or dampening positive emotions has also been found to be related to depressive symptoms (Bean et al., 2022; Burke et al., 2018). Furthermore, taking into account that emotion regulation can take place not only intrapersonally, but also in social contexts, suggests that investigating co-regulation patterns can add to the understanding of mental health in adolescence. For instance, co-rumination (excessive talking and dwelling) on negative emotions (Rose, 2002) with friends/peers has been found to be associated prospectively with depressive symptoms (Bastin et al., 2018) possibly via increased intrapersonal brooding rumination (Bastin et al., 2021).

The study focused on stress-related emotion regulation, but perceived stress was not assessed. However, everyday academic stress and interpersonal conflicts with peers and family members are consistently associated with negative daily mood (Bai et al., 2017). Difficulties in effectively altering negative mood or emotions triggered by these everyday events might contribute to depressive symptoms over time (Kovacs & Yaroslavsky, 2014). For instance, an ecological momentary assessment study conducted among adolescents (Rothenberg et al., 2019) found that increased daily sadness is associated with increased daily levels of depressive symptoms when the adolescent has more difficulty regulating their emotions on that day. Not only perceived stress as a proximal factor but also negative adverse childhood experiences as distal factors can lead to the development of depressive symptoms and increased levels of rumination later in life (Mansueto et al., 2021). However, in the present study, we did not assess distal factors, including adverse childhood experiences.

### Conclusion

Emotion regulation strategies have been extensively studied in relation to psychopathology or symptoms. However, empirical studies have predominantly focused on maladaptive strategies, particularly rumination. Furthermore, longitudinal studies have been even less likely to assess the protective effects of more adaptive strategies. This study explored the longitudinal connections between depressivesymptoms and both potentially adaptive and maladaptive strategies. It found comparable prospective associations between adaptive and maladaptive strategies with symptoms. Additionally, stable moderate and high symptoms groups, as identified by latent class analysis, showed a greater tendency to use maladaptive strategies and a reduced tendency to use adaptive strategies. Thus, targeted interventions for youth with elevated depressive symptoms are needed to enhance their emotion regulation skill and to teach adaptive emotion regulation strategies, such as reappraisal, in order to broaden their strategies repertoire and reduce reliance on strategies like rumination, which tend to amplify emotional experiences. The results also underscore the importance of identifying specific factors that can alleviate the adverse effects of maladaptive emotion regulation strategies and facilitate the adoption of more effective strategies to mitigate the impact of everyday stressors in adolescents.
